# Insect Bites

**Published:** 1914-06

**Authors:** 


**Insect Bites.** The Jour. of the N. A. R. D. recommends the following: To repel flies and other insects, Oil of sassafras, 1 part, oil of tar 2 parts, castor oil 5 parts. To relieve the pain and inflammatory reaction after insect bites occur, Oil of cloves 1, oil of lavender 1, balsam of fir 10, acetone 40, xylol 50, solution of formaldehyd 150.

